# Influence of Adiposity-Related Genetic Markers in a Population of Saudi Arabians Where Other Variables Influencing Obesity May Be Reduced

**DOI:** 10.1155/2014/758232

**Published:** 2014-11-17

**Authors:** Khalid K. Alharbi, Tom G. Richardson, Imran Ali Khan, Rabbani Syed, Abdul Khader Mohammed, Christopher R. Boustred, Tom R. Gaunt, Waleed Tamimi, Nasser M. Al-Daghri, Ian N. M. Day

**Affiliations:** ^1^Department of Clinical Laboratory Sciences, College of Applied Medical Sciences, King Saud University, P.O. Box 10219, Riyadh 11433, Saudi Arabia; ^2^MRC Integrative Epidemiology Unit, University of Bristol, Oakfield House, Oakfield Grove, Bristol BS8 2BN, UK; ^3^Bristol Genetic Epidemiology Laboratories, Department of Social and Community Medicine, University of Bristol, Oakfield House, Oakfield Grove, Bristol BS8 2BN, UK; ^4^Biomarkers Research Program, Biochemistry Department, College of Science, King Saud University, P.O. Box 2455, Riyadh 11451, Saudi Arabia; ^5^Prince Mutaib Chair for Biomarkers of Osteoporosis, Biochemistry Department, King Saud University, P.O. Box 2455, Riyadh 11451, Saudi Arabia; ^6^Department of Pathology and Laboratory Medicine (1122), King Fahad National Guard Hospital, King Saud Bin Abdulaziz University for Health Sciences, P.O. Box 22490, Riyadh 11426, Saudi Arabia

## Abstract

Large scale studies in Europeans have clearly identified common polymorphism affecting BMI and obesity. We undertook a genotype study to examine the impact of variants, known to influence obesity, in a sample from the Saudi Arabian population, notable for its profound combination of low mean physical activity indices and high energy intake. Anthropometry measures and genotypes were obtained for 367 Saudis, taken from King Saud University and Biomarker Screening Project in Riyadh (Riyadh Cohort). We observed large effect sizes with obesity for rs10767664 (*BDNF*) (OR = 1.923, *P* = 0.00072) and rs3751812 (*FTO*) (OR = 1.523, *P* = 0.016) in our sample and, using weighted genetic risk scores, we found strong evidence of a cumulative effect using 11 SNPs taken predominantly from loci principally affecting appetite (OR = 2.57, *P* = 0.00092). We used conditional analyses to discern which of our three highly correlated *FTO* SNPs were responsible for the observed signal, although we were unable to determine with confidence which best marked the causal site. Our analysis indicates that markers located in loci known to influence fat mass through increased appetite affect obesity in Saudi Arabians to an extent possibly greater than in Europeans. Larger scale studies will be necessary to obtain a precise comparison.

## 1. Introduction

In comparison with European populations, several factors may contrive to reduce the complexity of the trait of obesity in Saudi Arabia. The most important difference is in physical exercise. There is also a high wealth index and consequently high availability of high energy (carbohydrate and fat) diet. Local studies and international comparisons have shown that the Saudi population displays some of the lowest physical activity indices of any country [1, 2], severalfold lower than some nations. The very hot climate is a major inhibition for many physical activities. Many males drive a car directly from door to door between home and college (with petrol being cheaper than water and with space for cars, parking, and highways being generally abundant), and females either are driven (since females do not drive) or take a bus [3]. Furthermore, the culture does not encourage exercise in females [4]. Relatively less energy may be used for cold-provoked thermogenesis during the year. The absence of waist belts and concealment of figure may also serve to limit personal or social feedback which would have diverse impact through other cognitive pathways on eating behaviour. While smoking occurs, rates are relatively low especially in women [5] (although these rates are gradually increasing), so this source of impact on interindividual variance of weight may be more modest than in Europe or North America. Energy expenditure in the preparation of food is also low, because many households have a maid to help with many household tasks. Alcohol is not consumed whereas in European populations this is a significant additional source of variability of energy intake and weight gain (e.g., in vernacular, though a little misleadingly, the “beer belly” [6]). Taken together, these features would be predicted to combine to reduce the variables influencing obesity in Saudi Arabia, leaving a potentially greater impact particularly for determinants of appetite, amongst which genetic factors have an important role. Food intake and its rich composition are well established factors associated with obesity in Saudis [7].

The consequence would be that genetic variants determining appetite will have a greater effect both in determining individual weight and in accounting for the total population variance of obesity traits. This work represents a preliminary study of the magnitude of impact of genetic variants affecting appetite on obesity in the Saudi population.

## 2. Materials and Methods

### 2.1. Study Participants

Study participants were taken from two individual sources. 250 obese cases (BMI ≥ 30 kg/m^2^) were randomly selected from the Biomarker Screening Project in Riyadh (Riyadh Cohort), a capital-wide epidemiological study of over 17,000 consenting Saudis coming from different primary health care centres (PHCCs) in Riyadh, Saudi Arabia. Four subjects had missing BMI data leaving 246 subjects.

The other source provided BMI data for 207 subjects with Saudi Arabian ancestry who were enrolled at the College of Applied Medical Sciences from the male and female campus at King Saud University, although 3 subjects had missing values for BMI leaving 204 subjects.

We combined these two sets of participants together to obtain our sample of 450 subjects. We subsequently removed any subjects who had missing genotype data for any of the 11 SNPs we investigated, which left us with 367 individuals for our final sample. Ethical approval was obtained for both sources ((i) Biomarker Screening Project in Riyadh and (ii) College of Applied Medical Sciences). Written informed consent was obtained from all participants in the study.

### 2.2. DNA Extraction

Approximately 5 mL of whole blood was collected from each participant. 2 mL of the blood in EDTA-coated vacutainer was collected for genotyping and 3 mL of the serum was used for biochemical analysis. DNA was separated from whole blood using the blood genomic preparation minispin kit (GE Healthcare, USA) as described by Alharbi et al. [8]. DNA concentration and purity (260/280) were checked using NanoDrop Spectrophotometer.

### 2.3. Genotyping

Eleven genetic variants were chosen for this analysis based on previous studies in the literature which provided strong evidence of their association with obesity in European populations [9–11] (with the exception of rs9941349 which was found to be associated in a population of African descent [12]). The variants were rs10767664 (*BDNF*), rs3751812 (*FTO*), rs9939609 (*FTO*), rs9941349 (*FTO*), rs10938397 (*GNPDA2*), rs571312 (*MC4R*), rs2815752 (*NEGR1*), rs713586 (*RBJ*), rs543874 (*SEC16B*), rs7359397 (*SH2B1*), and rs2867125 (*TMEM18*).

### 2.4. Phenotyping

Subjects from the Riyadh Cohort were given a self-administered questionnaire to collect medical history and demographic information. Those with comorbidities that needed medical attention were excluded from the study. Participating subjects were requested to visit their assigned PHCCs after an overnight fast (>10 hours) for anthropometry and blood withdrawal. Anthropometry included height (to the nearest 0.5 cm) and weight (to the nearest 0.1 kg) utilizing a standardized measuring tape in cm.

For subjects from King Saud University, body weight in light clothing and height without shoes were measured to the nearest 0.1 kg and 0.1 cm, respectively. Weight was determined using a digital electronic scale, and height was measured using a standard steel strip stadiometer by trained personnel.

### 2.5. Statistical Analysis

Logistic regression was used to calculate odds ratios for obesity after categorizing lean controls (BMI < 25 kg/m^2^) and obese cases (BMI ≥ 30 kg/m^2^). Odds ratios comparing overweight individuals (25 kg/m^2^ < BMI < 30 kg/m^2^) with lean controls were also calculated. Weighted genetic risk scores were calculated using weights based on published effect sizes from European populations. Conditional analyses were performed by testing the association of SNP while adjusting for an index SNP in the same multivariate regression model. When undertaking this analysis we were working under the assumption that only one such variant is responsible for the association signal. BMI was log transformed on the log-10 scale to approximate univariate normality before conducting these analyses. Multiple testing was corrected for using the Bonferroni correction [13]. Stata 12.0 software (StataCorp, College Station, TX) was used for all statistical analyses. The *R* package metaplot was used to generate all figures.

## 3. Results

Descriptive characteristics for the 11 SNPs analysed are shown in Table 1. We found no evidence to suggest deviation of genotype frequencies from the Hardy-Weinberg equilibrium in our study population.  Table 2 shows variation across genotypes in BMI as a continuous trait in our study sample of 367 individuals with Saudi Arabian ancestry for whom we had both genotype and phenotype information. 218 of these individuals were taken from the Biomarker Screening Project in Riyadh (mean age: 24.52 years old (range: 18–30 years old)) and the other 149 were enrolled at King Saud University in Riyadh (mean age: 20.7 years old (range: 18–25 years old)).

**Figure 1:**
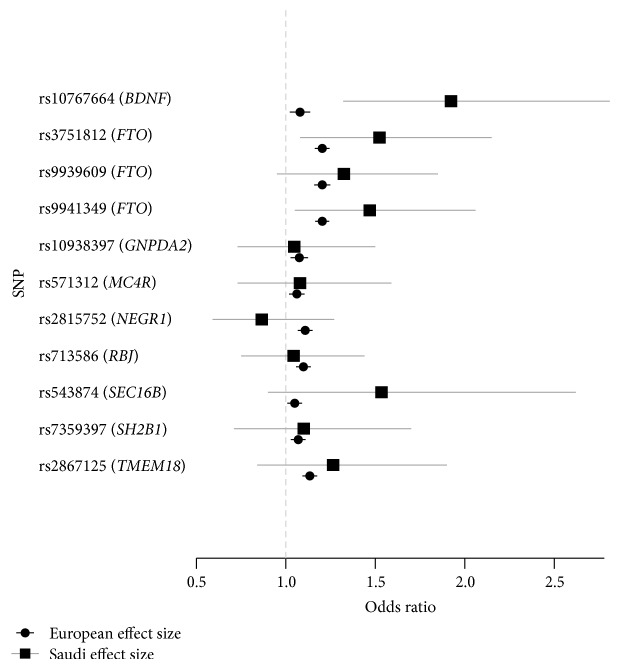
Forest plot comparing odds ratios for obese versus lean subjects between European and Saudi populations for 11 SNPs. rs3751812: effect size taken from NFBC1966 cohort in Finland [11], rs9939609: effect size taken from Frayling et al. study [10], rs9941349: effect size taken from Hassanein et al. study [12], and all others taken from GIANT consortium genome-wide association meta-analysis.

Odds ratios were calculated to examine the odds of obesity when compared to lean controls (236 versus 92) per risk allele for all 11 SNPs (Table 3). rs10767664 (*BDNF*) and rs3751812 (*FTO*) provided the strongest evidence of an association with obesity with odds ratios of 1.923 (CI: 1.32–2.81, *P* = 0.00072) and 1.523 (CI: 1.08–2.15, *P* = 0.016), respectively.  Figure 1 shows how the effects of each SNP observed in our study compare to estimates reported from high-powered European studies. Odds ratios were also calculated to examine the odds of being overweight when compared to lean controls (39 versus 92) per risk allele for all 11 SNPs (see Supplementary Table 1 in Supplementary Material available online at http://dx.doi.org/10.1155/2014/758232).

### 3.1. Genetic Risk Scores

Genetic risk scores were calculated for each subject for whom we had complete genotype information. rs9939609 and rs9941349 were removed from this analysis as including them would have invalidated the assumption concerning independence amongst variants due to the high LD with the other* FTO* SNP, rs3751812. We weighted our risk scores using published effect sizes from European cohorts (Table 4). Using the scores, we observed an odds ratio of 2.57 (95% CI: 1.448–4.562, *P* = 0.00092) calculating the odds of obesity (BMI ≥ 30 kg/m^2^) compared to lean controls (BMI < 25 kg/m^2^).

### 3.2. Conditional Analysis

Before conditioning on any other SNPs, rs3751812 and rs9941349 provided the strongest evidence that they were responsible for the observed association signal from this locus (*P* = 0.031 and *P* = 0.028, resp.). However, our conditional analysis did not provide sufficient evidence to discern which of these two variants was most tightly correlated with the true causal variant at this* FTO* locus, as the signal for both of them showed a major change when conditioned on the other (Table 5).

## 4. Discussion

We have conducted a pilot study to investigate the effect of 11 principally appetite-determining SNPs on obesity in a population of Saudi Arabians in highly powered European studies. Using weighted genetic risk scores, we provide strong evidence that these genetic variants are cumulatively associated with obesity in Saudi Arabians with the same direction of effect observed in Europeans (OR = 2.57, *P* = 0.00092).

Independently we observed that some of the central effects estimates for these SNPs were larger in our study compared to those found in European studies (Figure 1). For instance, we observed an odds ratio of 1.52 (95% CI: 1.08–2.15) for rs3751812 and 1.469 (95% CI: 0.95–1.85) for rs9939609 in our population of Saudi Arabians. In contrast, two of the best powered European studies which have analyzed the effect of these* FTO* variants on obesity are by Thomsen et al. [14] and Frayling et al. [10] who observed odds ratios of 1.42 (95% CI: 1.33–1.52) and 1.32 (95% CI: 1.26–1.39), respectively. However, larger study sizes will be needed to achieve high precision in these estimates.

We also observed an odds ratio of 1.92 per-additive alleles (95% CI: 1.32–2.81) for rs10767664 located in the* BDNF* gene, which despite the low sample size provided strong evidence of association with obesity (*P* = 0.0079, after adjusting for multiple comparisons). Furthermore, our 95% confidence interval did not contain the effect estimate observed in the GIANT consortium genome-wide association meta-analysis (OR = 1.079, 95% CI: 1.044–1.115 [9]). There has been some evidence to suggest that SNPs in the* BDNF *region may be implicated in dietary intake and snacking behaviour [15, 16]. This suggests that cultural differences in dietary eating habits may be responsible for the larger effect size we have observed in this study for our* BDNF *variant compared to those observed in European studies. It would therefore be worthwhile investigating this hypothesis in a future study and examining whether there is evidence of an interaction with dietary habits in this genotype-phenotype association.

We were interested in which of the three highly correlated* FTO* variants were most closely correlated with the biologically relevant variant at this locus or in discerning whether one of these variants themselves was responsible for the observed association signal. The observed association signal was weakest for the rs9939609 variant (*P* = 0.222) before conditioning on any other SNP, which meant this variant is the least likely to be correlated with the biologically relevant one based on this analysis. However, we were not able to discern with confidence which of the other two SNPs was the most tightly correlated with the true causal variant at this* FTO* locus. These results suggest that, concerning the signal with fat mass and obesity at this* FTO* locus, the Saudi population partially resembles what has previously been observed in populations of African descent rather than European. It should be noted that the* FTO* associations are believed to act through a long range chromatin effect on* IRX*3, a transcription factor highly expressed in hypothalamic weight-regulating centres [17]. Other appetite influencing variants certainly include* BDNF* and* MC4R*. While it is possible that appetite-determining genes might have a greater effect in Saudis and although they certainly contribute to the observed weight distribution, from a public health perspective it is also notable that physical activity does attenuate the effect of obesity genotypes [18].

### 4.1. Limitations

One of the populations in our study consisted of university students (mean age: 20.7 years old (range: 18–25 years old)) and therefore may not generalize to the whole population. Higher maternal educational status in Saudi Arabia has been observed to associate with higher physical activity levels [19]. Furthermore, young individuals may not display as much genotypic effect compared with middle-aged individuals who have developed a greater obesity phenotype. It is therefore possible that the genotypic effects may be even greater in other sectors of the population. As our study sample consisted predominantly of relatively young individuals (other portions of individuals in study were taken from the Biomarker Screening Project in Riyadh (mean age: 24.52 years old (range: 18–30 years old))), we were unable to extensively test this hypothesis.

As the sample is not large, the confidence intervals on our estimates of effect of appetite genotype alleles are wide. However, taken together, the results are consistent: the effects across genotype groups show consistent progressions, for example, for rs3751812, for BMI, TT > GT > GG with similar effect size between TT and GT as between GT and GG; and for unlinked independent gene loci known to influence appetite, the trends are mutually consistent.

It is most likely that factors such as dietary habits and physical activity have an impact on the effect size of the SNPs analysed in our study. We have attempted to quantify this with extended analyses, although, due to limited information on dietary intake for individuals (*N* = 104), we have lacked sufficient statistical power to report our results with confidence. Results of such analyses in a future study with a larger sample size should prove valuable.

## 5. Conclusions

Our analysis indicates that markers located in loci known to influence fat mass through increased appetite affect obesity in Saudi Arabians to an extent possibly greater than in Europeans. Larger scale studies will be necessary to obtain a precise comparison.

## Supplementary Material

Odds ratios were calculated to examine the odds of being overweight when compared to lean controls (39 versus 92) per risk allele for all 11 SNPs. Of these results, rs10938397 (*GNPDA2*) provided the only nominal evidence of association with obesity (OR: 1.622, CI: 1.00 – 2.62)

## Figures and Tables

**Table 1:** Summary of identified SNPs.

SNP	Change	Nearest gene	Chromosome	Position	MAF
rs10767664	A > T	*BDNF *	11	27725986	T = 0.256
rs3751812	G > T	*FTO *	16	53818460	T = 0.241
rs9939609	T > A	*FTO *	16	53820527	A = 0.355
rs9941349	C > T	*FTO *	16	53825488	T = 0.282
rs10938397	A > G	*GNPDA2 *	4	45182527	G = 0.346
rs2815752	G > A	*NEGR1 *	1	72812440	G = 0.301
rs571312	C > A	*MC4R *	18	57839769	A = 0.237
rs543874	A > G	*SEC16B *	1	177889480	G = 0.205
rs7359397	C > T	*SH2B1 *	16	28885659	T = 0.218
rs713586	T > C	*RBJ *	2	25158008	T = 0.446
rs2867125	T > C	*TMEM18 *	2	622827	T = 0.128

MAF: minor allele frequency. Chromosomal position according to dbSNP. MAF according to 1000 genomes project.

**Table 2:** Mean BMI across genotypes for 11 SNPs in a population of Saudi individuals.

SNP	Nearest gene	Effect allele	Other alleles	Mean BMI (SD) by genotype (other→effect)		
rs10767664	*BDNF *	A	T	26.105 (5.896)	29.519 (6.894)	31.385 (7.169)
rs3751812	*FTO *	T	G	29.524 (8.178)	30.637 (6.932)	31.096 (5.714)
rs9939609	*FTO *	A	T	30.074 (8.277)	30.270 (7.067)	30.845 (6.107)
rs9941349	*FTO *	T	C	29.556 (7.969)	30.431 (6.977)	31.333 (6.178)
rs10938397	*GNPDA2 *	A	G	30.674 (7.433)	30.004 (7.025)	30.466 (6.297)
rs2815752	*NEGR1 *	G	A	32.158 (7.559)	29.834 (6.887)	30.534 (7.233)
rs571312	*MC4R *	A	C	30.405 (7.148)	30.046 (7.203)	32.056 (6.561)
rs543874	*SEC16B *	A	G	29.639 (9.298)	28.877 (7.600)	30.785 (6.953)
rs7359397	*SH2B1 *	T	C	30.556 (7.343)	29.685 (6.481)	32.231 (7.721)
rs713586	*RBJ *	C	T	31.448 (7.799)	29.711 (6.767)	30.607 (7.121)
rs2867125	*TMEM18 *	C	T	30.518 (8.884)	29.276 (6.940)	30.849 (7.064)

Chr: chromosome; BMI: body mass index (kg/m^2^); SD: standard deviation.

**Table 3:** Odds ratios for obese versus lean subjects in a population of Saudi individuals for 11 SNPs.

SNP	OR (SE)	95% CI	*P* value
rs10767664 (*BDNF*)	1.923 (0.372)	1.32–2.81	0.00072
rs3751812 (*FTO*)	1.523 (0.266)	1.08–2.15	0.016
rs9939609 (*FTO*)	1.324 (0.227)	0.95–1.85	0.101
rs9941349 (*FTO*)	1.469 (0.254)	1.05–2.06	0.026
rs10938397 (*GNPDA2*)	1.047 (0.193)	0.73–1.50	0.801
rs571312 (*MC4R*)	1.079 (0.213)	0.73–1.59	0.701
rs2815752 (*NEGR1*)	0.865 (0.169)	0.59–1.27	0.456
rs713586 (*RBJ*)	1.043 (0.173)	0.75–1.44	0.799
rs543874 (*SEC16B*)	1.534 (0.418)	0.90–2.62	0.117
rs7359397 (*SH2B1*)	1.100 (0.246)	0.71–1.70	0.670
rs2867125 (*TMEM18*)	1.264 (0.264)	0.84–1.90	0.263

OR: odds ratio; SE: standard error; CI: confidence interval.

**Table 4:** Effect size of SNPs used for weighted genetic risk scores.

SNP	Nearest gene	Effect size
rs10767664	*BDNF *	0.19
rs3751812	*FTO *	0.36^*^
rs9939609	*FTO *	—
rs9941349	*FTO *	—
rs10938397	*GNPDA2 *	0.18
rs2815752	*NEGR1 *	0.13
rs571312	*MC4R *	0.23
rs543874	*SEC16B *	0.22
rs7359397	*SH2B1 *	0.15
rs713586	*RBJ *	0.14
rs2867125	*TMEM18 *	0.31

^*^rs3751812: effect size taken from NFBC1966 cohort in Finland [11]; all others taken from GIANT consortium genome-wide association meta-analysis [9].

**Table 5:** Conditional analysis on rs3751812, rs9939609, and rs9941349.

SNP	Effect estimate (SE)	*P* value	Effect estimate (SE)	*P* value	Effect estimate (SE)	*P* value	Effect estimate (SE)	*P* value
	Nonconditioned		Conditioned on rs3751812		Conditioned on rs9939609		Conditioned on rs9941349	
rs3751812	0.0164 (0.0075)	0.031			0.0334 (0.0151)	0.027	0.0045 (0.0280)	0.871
rs9939609	0.0093 (0.0077)	0.222	−0.0200 (0.0152)	0.192			−0.0142 (0.0135)	0.292
rs9941349	0.0166 (0.0075)	0.028	0.0122 (0.0279)	0.661	0.0282 (0.0133)	0.035		

SE: standard error.

## Abbreviations

BMI: Body mass index
OR: Odds ratio
SNP: Single nucleotide polymorphism
PHCC: Primary health care centre
EDTA: Ethylenediaminetetraacetic acid
DNA: Deoxyribonucleic acid
GIANT: Genetic investigation of anthropometric traits
MAF: Minor allele frequency
SE: Standard error.
