# Supplementary material for: Influence of Adiposity-Related Genetic Markers in a Population of Saudi Arabians Where Other Variables Influencing Obesity May Be Reduced
Source: Dis Markers. 2014 Nov 17;2014:758232. doi: 10.1155/2014/758232 (PMC4251424; doi:10.1155/2014/758232)
Supplement: Supplementary file 1 — Odds ratios were calculated to examine the odds of being overweight when compared to lean controls (39 versus 92) per risk allele for all 11 SNPs. Of these results, rs10938397 (GNPDA2) provided the only nominal evidence of association with obesity (OR: 1.622, CI: 1.00 – 2.62) [file 758232.f1.pdf]

Supplementary Table 1: Odds Ratios for overweight vs lean subjects in a population of Saudi individuals for 11 SNPs

**SNP**	**OR (SE)**	**95% CI**	**P-value**
rs10767664 (*BDNF*)	1.029 (0.264)	0.62 – 1.70	0.912
rs3751812 (*FTO*)	1.381 (0.338)	0.86 – 2.23	0.187
rs9939609 (*FTO*)	1.335 (0.326)	0.83 – 2.16	0.237
rs9941349 (*FTO*)	1.295 (0.318)	0.80 – 2.10	0.293
rs10938397 (*GNPDA2*)	1.622 (0.399)	1.00 – 2.62	0.049
rs571312 (*MC4R*)	1.052 (0.296)	0.61 – 1.82	0.856
rs2815752 (*NEGR1*)	1.189 (0.342)	0.68 – 2.09	0.548
rs713586 (*RBJ*)	1.286 (0.331)	0.78 – 2.13	0.329
rs543874 (*SEC16B*)	1.496 (0.589)	0.69 – 3.24	0.307
rs7359397 (*SH2B1*)	0.867 (0.288)	0.451 – 1.66	0.668
rs2867125 (*TMEM18*)	1.187 (0.359)	0.66 – 2.15	0.571

- OR – Odds Ratio, SE – Standard Error, CI – Confidence Interval
